# Alternative Responses to Predation in Two Headwater Stream Minnows Is Reflected in Their Contrasting Diel Activity Patterns

**DOI:** 10.1371/journal.pone.0093666

**Published:** 2014-04-01

**Authors:** Wilbert T. Kadye, Anthony J. Booth

**Affiliations:** Department of Ichthyology and Fisheries Science, Rhodes University, Grahamstown, South Africa; The Australian National University, Australia

## Abstract

Animals exhibit diel periodicity in their activity in part to meet energy requirements whilst evading predation. A competing hypothesis suggests that partitioning of diel activities is less important because animals capitalise on opportunity. To test these hypotheses we examined the diel activity patterns for two cyprinid minnows, chubbyhead barb *Barbus anoplus* and the Eastern Cape redfin minnow *Pseudobarbus afer* that both occur within headwater streams in the Eastern Cape, South Africa. Chubbyhead barbs exhibited consistent nocturnal activity based on both field and laboratory observations. Due to the absence of fish predators within its habitat, its nocturnal behaviour suggests a response to the cost associated with diurnal activity, such as predation risk by diving and wading birds. In contrast, redfin minnows showed high diurnal activity and a shoaling behaviour in the wild, whereas, in the laboratory, they showed high refuge use during the diel cycle. Despite their preference for refuge in the laboratory, they were diurnally active, a behaviour that was consistent with observations in the wild. The diurnal activity of this species suggests a response to the cost associated with nocturnal activity. Such a cost could be inferred from the presence of the longfin eel, a native predator that was active at night, whereas the daytime shoaling behaviour suggests an anti-predator mechanism to diurnal visual predators. The implications of these findings relate to the impacts associated with the potential invasions by non-native piscivores that occur in the mainstem sections. Diurnal activity patterns for redfin minnows, that are IUCN-listed as endangered, may, in part, explain their susceptibility to high predation by visual non-native piscivores, such as bass and trout. In contrast, the nocturnal habits of chubbyhead barbs suggest a probable pre-adaptation to visual predation. The likelihood of invasion by nocturnally-active sharptooth catfish *Clarias gariepinus*, however, may compromise this prior advantage.

## Introduction

It is assumed that diel activity patterns have evolved in response to both exogenous stimuli and endogenous biological constraints [1]–[4]. Exogenous stimuli, including predation, prey dynamics, competition and ambient environmental conditions, are considered to be the selective ecological drivers for the contrasting effects of relative risk and resource use within a particular environment [5]. Adaptive responses to such stimuli are reflected by specialisation towards either diurnal or nocturnal use of available space and resources. Diel activity patterns are therefore considered to be a temporal axis that can facilitate niche segregation among potential competitors [6]–[8] and coexistence within predator-mediated systems [9].

Activity patterns in prey fishes exhibit particular diel rhythms that are reflected by nocturnal, diurnal or crepuscular habits [10], [11]. For stream fishes, such diel activity patterns are considered to be a response to both risk, such as the differential predation effects either by piscivorous predators in deep water or by wading and diving terrestrial predators in shallow water [12], and potential reward from foraging opportunities [13]. Prey fishes therefore select alternative patches that will yield minimum daily food requirements against negative risk associated with predation and competition [14]. Such behavioural responses by prey fish species would manifest in restrictions of their activities to periods when there is a perceived low prohibitive risk and a high profitable foraging opportunity [13], [15]. Studies have also alluded to the importance of local habitat features, such as cover, depth and habitat complexity in conferring both refuge and foraging space [16], [17]. Commonly, analyses of the relationships between population dynamics and physical attributes are used to infer habitat preference or avoidance [18], which suggests the importance of such physical attributes to both the temporal and spatial patterns in resource use among stream fishes [16].

Understanding diel patterns of stream fishes, particularly those that are prey fishes, is critical because of the increasing threat posed by the establishment of non-native piscivorous fishes. Since diurnal-to-nocturnal synchrony is considered to be an adaptive response that has developed over a longer evolutionary scale, there is need to understand how diel activity patterns relate to susceptibility of native species to invasion impacts. While many studies have provided insights on the spatial aspects of niche partitioning where potential impacts are likely to be high within invaded zones [19], [20], it is not clear how the temporal aspects related to the perceived physiological and adaptive traits of native prey species contributes to our understanding of their persistence, or lack thereof, in invaded habitats. For example, recent studies have reported the sympatric occurrence of both non-native piscivorous invaders and native prey fish [21], which suggest that the behavioural adaptation traits, including diel activity patterns, may play an important role in reducing potential impacts. Recent studies have revealed the role of diel adaptations in facilitating predator avoidance in invaded systems [22].

Ecological theory suggests that animals are evolutionarily constrained to their activity patterns, which ultimately fixes temporal partitioning, rendering them less amenable to manipulation [23]–[25]. This hypothesis suggests that animals will show fixed diel patterns due to specific physiological and adaptive traits that would result in sub-optimal performance during the alternate diel period [26], [27]. However, certain species have been found to exhibit flexibility in their adaptations to activity patterns [5], [28], [29]. Several studies on freshwater fishes have provided evidence for such plasticity in activity patterns [15], [30], [31]. This alternative hypothesis suggests that temporal partitioning is less important as animals tend to maximise on opportunity, and animals would therefore be able to exhibit flexibility in their diel activity [32].

We test these hypotheses by comparing the diel activity patterns of two cyprinid minnows, the chubbyhead barb *Barbus anoplus* and the Eastern Cape redfin minnow *Pseudobarbus afer*. Both species occur within headwater tributaries of rivers in the Eastern Cape, South Africa. These two minnows are an integral component of the ecological functioning and food webs within headwater streams. There are, however, serious concerns over the invasion of their habitats by non-native piscivores, such as sharptooth catfish *Clarias gariepinus*, largemouth bass *Micropterus salmoides* and smallmouth bass *M. dolomieu* that occur within the mainstem sections of many rivers [33]. We relate our findings to the responses that have been observed in *B. anoplus* and *P. afer* to the invasion of their habitats by non-native predators. We therefore hypothesize that diel activity patterns of these two species would reflect their potential susceptibility to different non-native invaders.

## Methods

### Ethics statement

This study was conducted in accordance with the guidelines for the use of animals in research for South Africa. Research on both chubbyhead barbs *Barbus anoplus* and redfin minnows *Pseudobarbus afer* was approved by the Eastern Cape's Department of Economic, Development and Environmental Affairs through permit numbers CRO 67/13CR, CRO 68/13CR and CRO 69/13CR. Research within the Groendal Wilderness Area for redfin minnows was approved by the Eastern Cape's Parks and Tourism Agency through permit number RA 0151 and the Rhodes University Ethics committee. All animals were sampled using non-destructive minnow traps and through seine netting.

### Study species

The chubbyhead barb, *B. anoplus*, is common in many rivers of the region and is the most widespread minnow in South Africa [34]. It is particularly abundant within headwater streams [35] and is listed as a species of least concern under the IUCN list of threatened taxa [36]. Anecdotal evidence suggests that it occurs in sympatry with non-native piscivores, such as largemouth and smallmouth bass and rainbow trout *Onchorynchus mykiss* in certain habitats. The Eastern Cape redfin minnow *P. afer*, by contrast, is listed as endangered [36], and is restricted to headwater streams that drain the Cape Fold Mountains. *Pseudobarbus* spp. are considered to be threatened primarily by non-native piscivorous predators. Both species are persistent in headwater streams with their population dynamics driven primarily by recruitment [35]. We investigated the diel activity patterns for these two species using both field and laboratory experiments.

### Field experiments

To assess diel activity, we conducted sampling for both chubbyhead barbs and redfin minnows between May and July 2013. Chubbyhead barbs were sampled in the headwaters of the Mankazana River, a tributary of the Koonap River that is a major tributary of the Great Fish River. No other fish species were found in the sampled stream. Redfin minnows were sampled in the Waterkloof River, a tributary of the Swartkops River within the Groendal Wilderness Area. Two other native fish species, Cape kurper *Sandelia capensis* and longfin eel *Anguilla mossambica* also occurred in the sampled stream.

To estimate abundance, minnow traps (50 cm long by 25 cm diameter and 3 cm diameter opening, with 2 mm mesh), baited with trout pellets, were randomly positioned in different habitats of each stream. The traps were deployed from 07:00 to 16:00 hrs and 18:00 to 06:00 hrs for diurnal and nocturnal observations, respectively. The sampled stream consisted of a series of pools and riffles. We deployed 20 traps for each sampling occasion in new and different localities. Each trap was positioned at least 30 m apart to minimise resampling the same fish. Sampling was conducted over three consecutive days and nights. The microhabitat around each trap was assessed based on water depth (cm), dominant substratum and the presence or absence of bank vegetation. Substratum composition was categorised based on a modified Wentworth scale [37] as coarse gravel (<6 cm), cobble (6–25 cm), boulder (25 cm–100 cm) and bedrock (>1 m). To determine the presence and diel patterns of predatory fish, six double-ended fyke nets were set randomly and monitored over the same three consecutive days and nights. The fyke nets were set between 07:00 to 16:00 hrs and 18:00 to 06:00 hrs for diurnal and nocturnal observations, respectively. Both minnow traps and fyke nets were observed between 16:00 to 18:00 hrs and 06:00 to 08:00 hrs for the day and night captures, respectively. All fish that were captured were identified, measured (standard length) and released back into the river alive.

### Laboratory experimental procedure

Chubbyhead barbs and redfin minnows were captured by seine netting and transported to the laboratory in oxygenated tanks. The batches for the two species were left to acclimatise in holding tanks (90 cm×32 cm×40 cm in length, width and height, respectively) in the laboratory for a period of at least four weeks. The laboratory was designed to simulate a 12 hour day and night photoperiod using timer-controlled fluorescent lights. The day period was therefore illuminated from 00:00 to 12:00 hrs, whereas the night period commenced from 12:00 to 00:00 hrs. This illumination cycle allowed for the observations of both diurnal and nocturnal activity during the course of a working day. We maintained the fish in clean filtered water. Water temperature was maintained at 20°C and dissolved oxygen was kept at saturation level. The fish were fed daily on standard commercial aquarium fish flakes.

After the acclimation period, the fish were size-sorted into two groups of small (<40 mm TL) and large fish (>40 mm TL) corresponding to immature and mature individuals respectively [38], [39], after which they were transferred to their experimental tanks. Both species reach sexual maturity at approximately 40 mm TL [38], [39]. Experimental tanks comprised of 20 identical units each measuring 30 cm×23 cm×24 cm in length, width and height, respectively. Each tank, which contained an undergravel bed with an air-lift oxygenation system, was divided into three equal areas; pipe refuge, “grass” refuge, and open water. The pipe refuge was a half-cylinder pipe (110 mm in diameter and 150 mm long). The grass refuge comprised of polythene strips (4 mm in width and 200 mm long) woven into a 10 cm^2^ polythene grid that was secured to the false-bottom and covered with gravel to allow the strips to float freely above the substrate. Both refuge types were placed adjacent to each other at the back of the tank, with the front section of the tank constituting the open-water section. Each experimental tank was enclosed with black polythene cover on the sides with a small flap cut into the polythene that could be lifted for making observations. All tanks were positioned at eye-level in the laboratory and illuminated from above.

We hypothesized that, for each species, diel activity patterns would differ between size classes (small versus large fish) and in the presence or absence of conspecifics (individuals versus being in a group). To test these hypotheses, we conducted a factorial experiment on both size class and conspecifics whereby each of the four size class × conspecific treatments had five replicates randomly allocated to the 20 experimental tanks. The single fish treatments for both small (S1) and large (L1) fish comprised of one individual, whereas the grouped fish treatments comprised of three individuals for both small (S3) and large (L3) fish. Fish that were transferred to the experimental tanks were allowed to acclimate for a period of two days after which observations on diel activity patterns commenced. Fish were fed daily with the food dispensed evenly throughout each tank. Observations were made twice for each of the light and dark periods per day. To minimise the effect of the feeding on activity rhythms, observations were made before the fish were fed and at least two hours after feeding. The experimental trial was duplicated with each trail session consisting of four consecutive days and nights. Each inter-trial period lasted for one week during which the experimental tanks were cleaned and their water replaced. The treatments were then randomly re-assigned to the experimental tanks before the second trial. A total of 40 fish were used during each trial, and no fish was used more than once. We made observations on habitat choice and activity during each diel cycle. For habitat choice, we recorded each individual fish in relation to their occurrence in either pipe, grass or in open water within each experimental tank. Fish activities were recorded into three categories as being inactive (motionless on the bottom), slightly active (hovering in mid-water) and active (swimming in mid-water), and these categories were coded as 1, 2 and 3, respectively.

### Statistical analysis

For the field experiments, relative fish abundance (number of fish per trap) was assessed between day and night photoperiods and the physical habitat attributes (depth, substratum composition and presence/absence of vegetation cover). Relative fish abundance was modelled with a Poisson generalised linear mixed models (GLMM) and log-link function after examining for possible overdispersion [40]. To standardise catch rate, trap soak time was included as an offset term in the model. Furthermore, we used the GLMM models to compare whether relative abundance was depth-dependent between each of the day and night photoperiods by including the interaction between depth and photoperiod into the model. Model validity was assessed by applying Kolmolgorov-Smirnov and Cramer-von Mises [41] goodness-of-fit tests on the cumulative residuals.

For the laboratory experiments, we used frequency and activity of fish as response variables to examine the effect of photoperiod (day and night), habitat choice (pipe, grass and open water), size (small and large) and conspecifics (individual versus grouped fish). The frequency data, being multinomial response data, were assessed with Poisson GLMM with a log-link function. Activity was assessed with a linear mixed model (LMM). For the GLMM and LMM models, we used Laplace and restricted maximum likelihood (REML) approximations, respectively, for the maximum likelihood estimation of parameters [40]. The statistical significance of the fixed effects were tested using Wald χ^2^ tests.

Models were developed using best subset modelling [42]. In total, the candidate models considered were 56 models for the field-based abundance data, 19 models for the laboratory-based activity data, and 38 models for the laboratory-based frequency data. For the field experiments, the model for each species included photoperiod, depth, substratum and vegetation as fixed effects, and photoperiod × depth and substratum × vegetation as interaction terms. For the laboratory experiments, analysis of the frequency model for each species included photoperiod, choice, conspecifics and size as fixed effects, and photoperiod × choice × conspecifics as interaction terms. Analysis of the activity model for each species included photoperiod, conspecifics and size as fixed effects, and photoperiod × conspecifics × size as the interaction terms. The candidate model sets were developed from a fully saturated model that included all terms and their interactions. Akaike's Information Criterion (AIC) was used to compare each model's goodness-of-fit. We identified models with the lowest AIC as the most parsimonious, and evaluated each model's relative strength based on their Akaike weights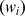 that is calculated from 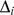, the difference between the AIC for the best model and all other models in the candidate set. Only those models with 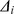
≤2 were considered to have substantial support from the data [42]. All analyses were conducted within R [43] using the packages *lme4* [44] and *MuMIn* [45].

## Results

**Figure 1:**
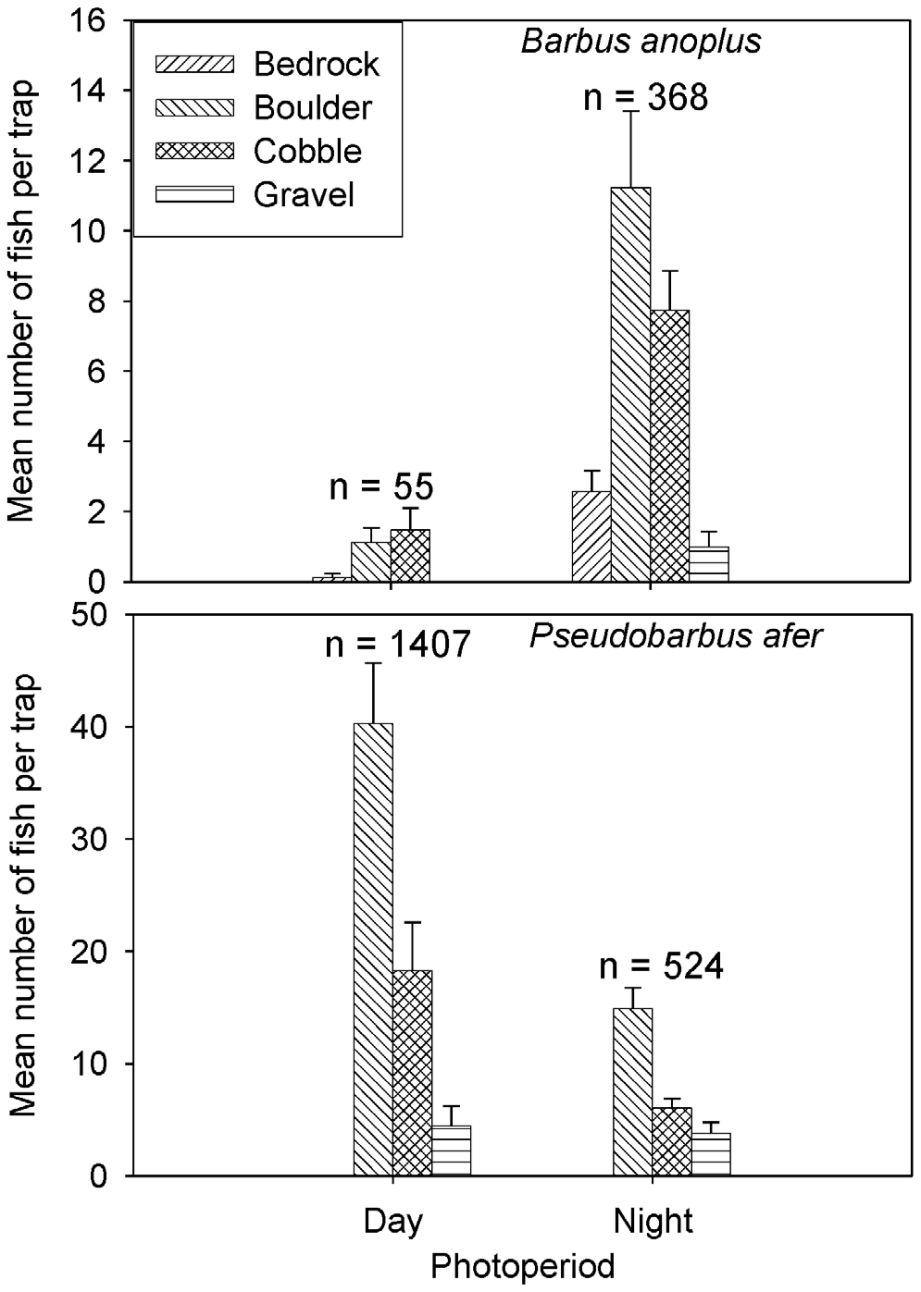
The number (mean ± standard error) of fish caught in minnow traps in relation to different substratum categories during field experiments for chubbyhead barb *Barbus anoplus* and Eastern Cape redfin minnow *Pseudobarbus afer* during day and night. Sampling was conducted over three consecutive days and nights for each species.

A total of 423 chubbyhead barbs and 1931 redfin minnows were collected during the field experiments (Figure 1). No other fish species were captured in habitats with chubbyhead barbs. In contrast, a total of seven longfin eels, *Anguilla mossambica*, which are known predators, were captured using fyke nets at night in habitats with redfin minnows. The GLMMs, with 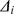
≤ 2, for chubbyhead barbs abundance explained between 82 and 98% of the variation in the data (Table 1). Photoperiod, depth and substratum were the significant variables that explained chubbyhead barb abundance in all the models. Based on the most parsimonious model, chubbyhead barb abundance differed between photoperiods (χ^2^ = 124.2, df = 1, *p*<0.001). Furthermore, chubbyhead barb showed strong nocturnal activity whereby 87% (n = 368) of the fish were captured at night, whereas daytime captures accounted for 13% (n = 55) of the catches (Figure 1). For redfin minnows, the best ranked models for its abundance explained 80% of the variation (Table 1). The best ranked models included photoperiod, substratum and depth together with the interaction between depth and photoperiod as the significant variables explaining their relative abundance (Table S1). Similar to chubbyhead barbs, redfin minnow relative abundance differed between photoperiods (χ^2^ = 594.5, df = 1, *p*<0.001) based on the most parsimonious model. However, in contrast to the abundance patterns observed for chubbyhead barbs, redfin minnows exhibited a shoaling behaviour, and showed high diurnal activity pattern whereby 73% (n = 1407) of the fish were captured during the day compared to 27% (n = 524) during the night. For both species, the highest number of captured fish was significantly associated with boulders and cobbles (Figure 1).

**Table 1:** Analysis of Poission generalized linear mixed models indicating the null and best-subset models describing the relative abundance patterns of chubbyhead barb *Barbus anoplus* and Eastern Cape redfin minnow *Pseudobarbus afer* that were captured in the wild in relation to photoperiod and physical habitat variables.

		*AIC*	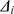	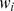	*R^2^*
*Barbus anoplus*	Intercept	740.9	299.5	0.00	0.00
	Intercept + **Depth**+**Photoperiod** + **Substratum** + Vegetation + Substratum ×Vegetation	414.4	0.00	0.14	0.98
	Intercept + **Depth** + **Photoperiod** + **Substratum** + Vegetation + Substrate×Vegetation + Depth × Photoperiod	414.7	0.28	0.12	0.98
	Intercept + **Depth** + **Photoperiod** + **Substratum** + Vegetation	442.9	1.44	0.07	0.82
	Intercept + **Depth** + **Photoperiod** + **Substratum** + Vegetation + Depth × Photoperiod	443.2	1.75	0.06	0.82
*Pseudobarbus afer*	Intercept	1537.2	579.00	0.00	0.00
	Intercept + **Depth** + **Photoperiod** + **Substratum** + **Depth** × **Photoperiod**	961.2	0.00	0.33	0.80
	Intercept + **Depth** +**Photoperiod** + **Substratum** + Vegetation + **Depth** × **Photoperiod**	963.2	1.97	0.12	0.80

In all models, catch rate was standardised by time with the inclusion of soak time as an offset term. AIC  =  Akaike's Information Criterion, 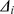 =  the difference in AIC values between the candidate model and the most parsimonious model with the lowest AIC, 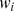 =  the model weight in relative to all models assessed, and *R^2^* =  the pseudo-coefficient of determination. Significant effects (α<0.05) are indicated in bold.

**Figure 2:**
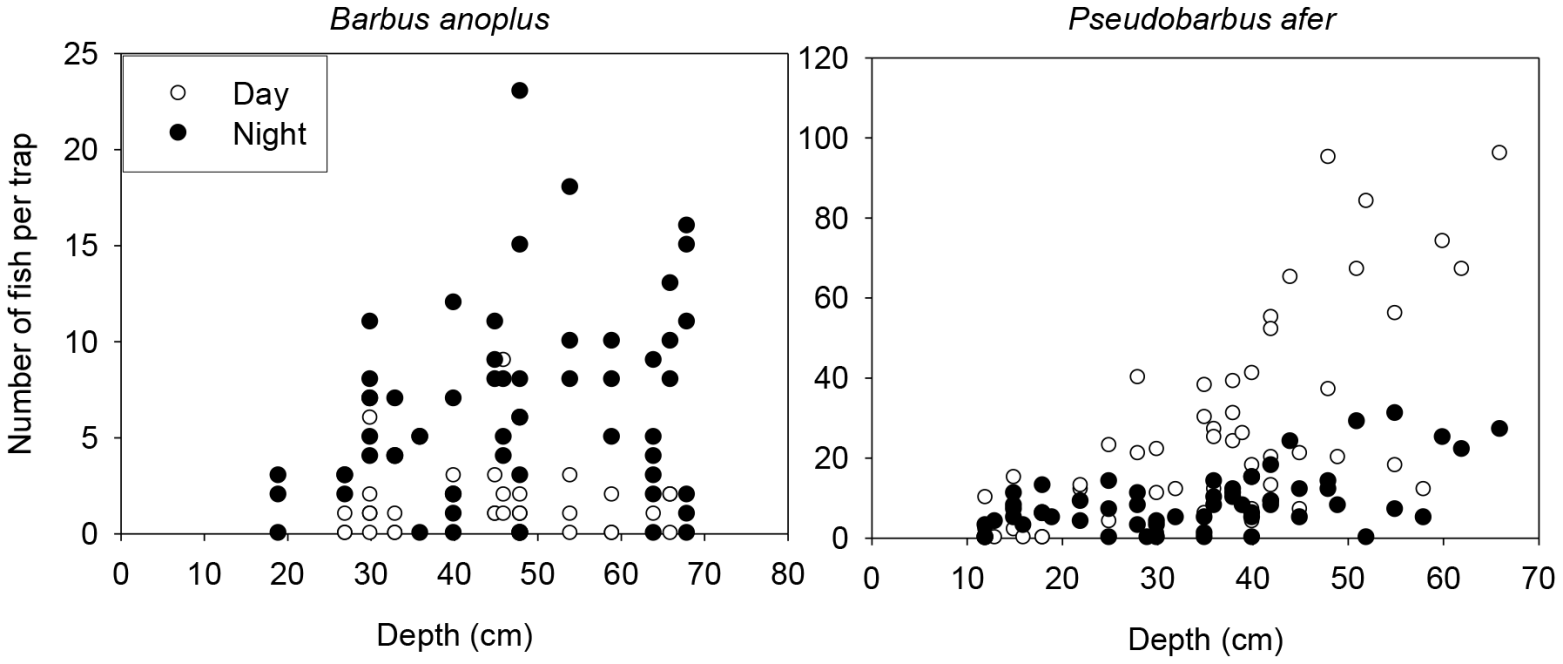
The relationship between fish abundance and depth during day and night for chubbyhead barb *Barbus anoplus* and Eastern Cape redfin minnow *Pseudobarbus afer* based on field experiments.

Chubbyhead barb abundance showed a moderate increase with depth (χ^2^ = 4.7, df = 1, p = 0.03). However, there was no interaction between depth and photoperiod (χ^2^ = 1.6, df = 1, p>0.05) with the results indicating a weak positive correlation between abundance and depth at night (Spearman's rank correlation, *r* = 0.23, p>0.05) and no correlation for daytime observations (Spearman's rank correlation, *r* = 0.08, p = 0.49). By contrast, redfin minnow abundance was significantly influenced by depth (χ^2^ = 99.8, df = 1, p<0.001) together with having a significant interaction between depth and photoperiod (χ^2^ = 30.2, df = 1, p<0.001), indicating that the relationships between depth and abundance were different between day and night. Although redfin minnow abundance increased with depth for each of the photoperiods (Figure 2), this relationship was stronger (Spearman's rank correlation, *r* = 0.70, p<0.001) during the day compared to night (Spearman's rank correlation, *r* = 0.57, p<0.001).

**Figure 3:**
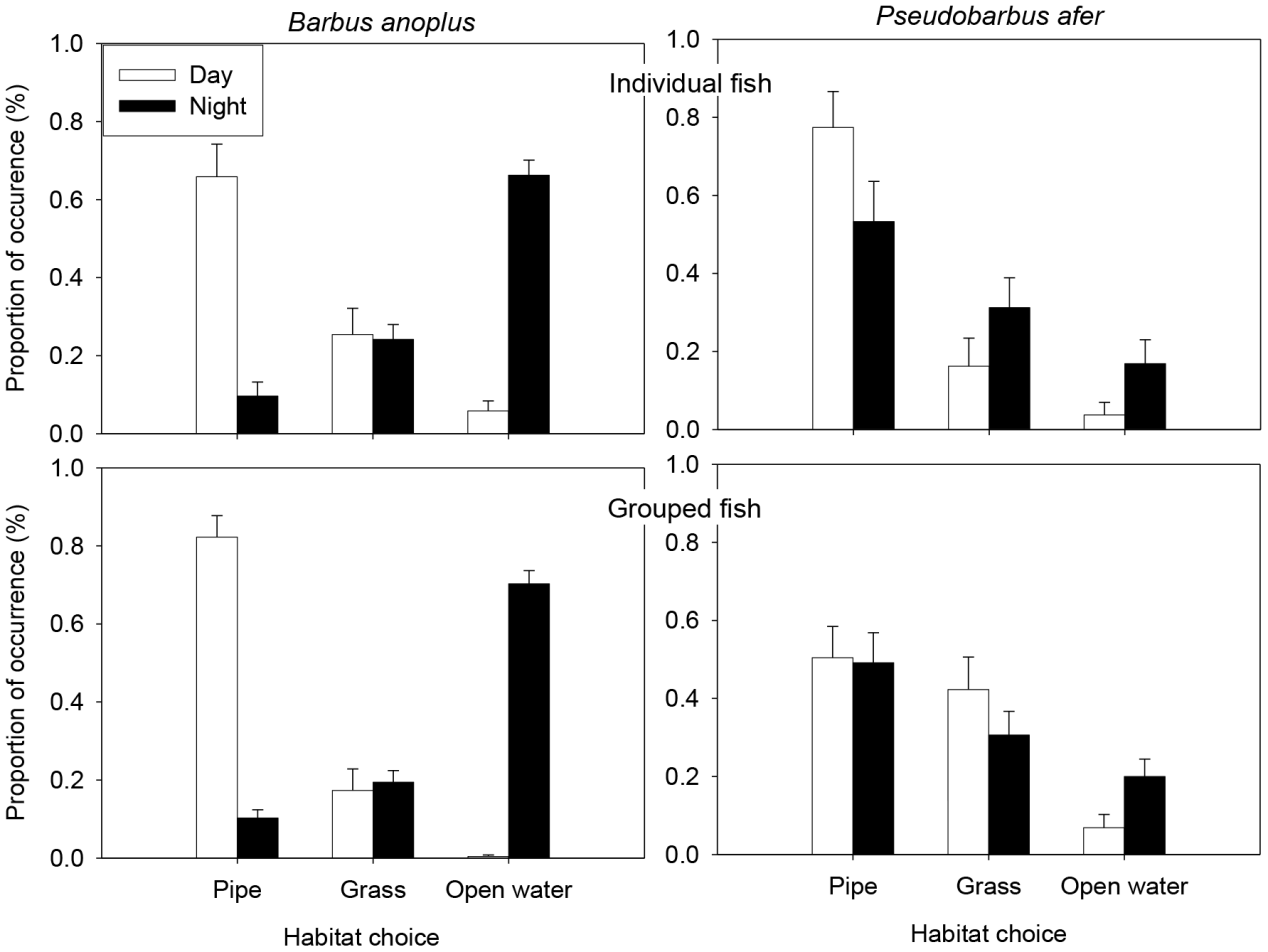
Laboratory experiments showing the proportion (mean ± standard error) of individual and grouped fish associated with pipe, grass and open water habitats during day and night for chubbyhead barb *Barbus anoplus* and Eastern Cape redfin minnow *Pseudobarbus afer*.

In the laboratory, chubbyhead barbs exhibited a marked temporal difference in habitat association. The best ranked GLMMs showed that the fixed effects (photoperiod, choice and conspecifics) and their interactions were most important in explaining chubbyhead barbs frequency of habitat association (Table 2). Fish showed high association with pipe refuge during the day and open water at night (Figure 3). Inference from the most parsimonious model revealed an interaction between photoperiod and habitat choice (χ^2^ = 7.5, df = 2, p = 0.02) (Table 2), indicating that habitat selection was dependent on photoperiod. Specifically, the conspecifics × open water interaction indicated that grouped fish utilised open water in higher frequencies than individuals (Table S2, Figure 3). Furthermore, there were differences in habitat association based on presence or absence of conspecifics (χ^2^ = 189.2, df = 1, p<0.001), with the results indicating that individual fish were associated with grass refuge in relatively higher proportions compared to grouped fish (Figure 3). For redfin minnows, the best ranked GLMMs showed that habitat associations were explained by the presence or absence of conspecifics (Table 2). Based on the most parsimonious model, redfin minnows were associated with the different refuge type equally throughout the diel cycle (χ^2^ = 4.72, df = 2, p = 0.09) (Figure 3, Table S2). Nevertheless, redfin minnows exhibited significant conspecific effects (χ^2^ = 69.0, df = 1, p<0.001) with individual fish preferring pipe refuge, whereas, grouped fish were associated with both pipe and “grass” refugia during the day (Figure 3). Furthermore, we found that redfin minnows were associated with open water at night rather than during the day.

**Table 2:** Analysis of GLMMs and LMMs on laboratory observations indicating the null and best-subset models describing either the frequency of observations or activity levels within different refugia for chubbyhead barb *Barbus anoplus* and Eastern Cape redfin minnow *Pseudobarbus afer* in relation to photoperiod, choice of refuge, size, presence of conspecifics.

	Frequency	*AIC*	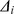	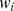	*R^2^*
*Barbus anoplus*	Intercept	3098.5	173.2	0.00	0.00
	Intercept + **Choice** + **Conspecifics** + **Photoperiod** + **Choice×Conspecifics** + **Choice×Photoperiod** + **Conspecifics×Photoperiod** + Choice×Conspecifics×Photoperiod	179.0	0	0.34	0.24
	Intercept + **Choice** + **Conspecifics** + **Photoperiod** + **Choice×Conspecifics** + **Choice×Photoperiod**+**Conspecifics×Photoperiod**	179.3	0.37	0.28	0.24
	Intercept + **Choice** + **Conspecifics** + **Photoperiod** + Size + **Choice×Conspecifics** + **Choice×Photoperiod** + **Conspecifics×Photoperiod** + Choice×Conspecifics×Photoperiod	180.7	1.73	0.14	0.24
*Pseudobarbus afer*	Intercept	2221.2	60.3	0.00	0.00
	Intercept + Choice + **Conspecifics**	173.8	0	0.16	0.11
	Intercept + Choice + **Conspecifics** + Size	174.1	0.28	0.14	0.12
	Intercept + **Conspecifics** + Size	174.8	0.96	0.10	0.11
	Intercept + **Conspecifics**	175.1	1.22	0.09	0.11
	Intercept + Choice + **Conspecifics** + Photoperiod + Size	175.6	1.79	0.07	0.12

The frequency models were Poisson, while activity models were Guassian. AIC  =  Akaike's Information Criterion, 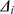 =  the difference in AIC values between the candidate model and the most parsimonious model with the lowest AIC, 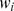 =  the model weight in relative to all models assessed, and *R^2^* =  the pseudo-coefficient of determination. Significant effects (α<0.05) are indicated in bold.

**Figure 4:**
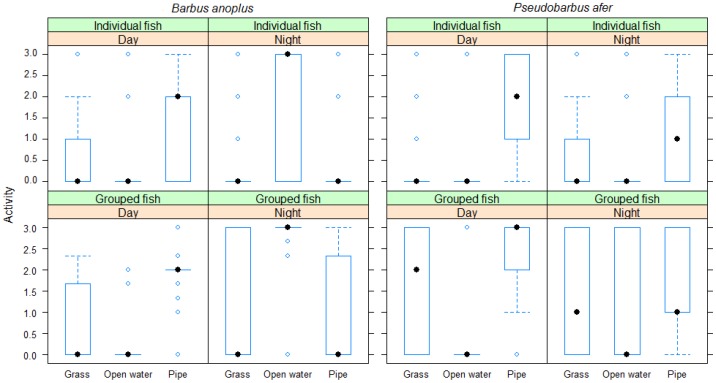
Laboratory experiments indicating the activity patterns (median, 25% and 75% inter-quartiles) of individual and grouped fish associated with pipe, grass and open water habitats during day and night for chubbyhead barb *Barbus anoplus* and Eastern Cape redfin minnow *Pseudobarbus afer*.

Activity patterns in chubbyhead barbs mirrored the field observations, with high fish activity being observed at night in open water for both individual and grouped fish (Figure 4). The best fitted model revealed that fish activity was influenced only by photoperiod (χ^2^ = 2663.2, df = 1, p<0.001). In comparison, redfin minnows' activity was influenced by both photoperiod and conspecific effects (Table 2). Although redfin minnows were associated with pipe refugia throughout the diel cycle, they had high diurnal activity (χ^2^ = 11.2, df = 1, p<0.001) (Figure 4). The activity rate of redfin minnows was higher in the presence of conspecifics (χ^2^ = 32.2, df = 1, p<0.001).

## Discussion

### Diel activity patterns in minnows

The two species in this study exhibited contrasting diel activity patterns. Based on the field experiments, we found that chubbyhead barbs were nocturnal, whereas, redfin minnows showed high diurnal activity. The results of this study broadly supported the hypothesis that the two species have fixed diel activity patterns. Nevertheless, their contrasting diel activities suggest that they exhibited differences to risk perception and foraging opportunity in response to the proximate cues within their particular habitats. Studies have shown that the preferred period of activity by animals is associated with maximising energy gain while minimising predation risk [13]. For chubbyhead barbs, their nocturnal behaviour suggests a response to the costs associated with daytime activity. Because nocturnal activity is considered to be inefficient for visual foraging [11] particularly for stream-dwelling minnows [46], and due to the absence of fish predators within its habitat, the nocturnal habit of chubbyhead barbs suggests a response to visual terrestrial predators, such as diving and wading birds. Nocturnal investment by fishes usually reflects a perceived high foraging cost, primarily due to predation risk, during the day rather than at night [26]. The evolution of a nocturnal lifestyle is therefore believed to have been driven by visual diurnal predators [5] and is common in fish [23], [47], [48], and has been observed for other taxa, including aquatic insect larvae [49], [50] and zooplankton [51].

Diurnal activity by redfin minnows suggests a response to the relative costs associated with nocturnal activity. Although the cost of nocturnal activity could be inferred from the presence of longfin eel *A. mossambica* that was active at night in habitats with the minnows, visual terrestrial predators, such as birds, could potentially be associated with the cost of diurnal activity for this species. Furthermore, the sympatric occurrence of redfin minnows with *S. capensis* suggests potential for resource competition. Studies show that when animals are faced with different costs associated with satisfying minimum energy requirements, due to either predation or competition, they often learn to discriminate between these costs and individual risks [52]. Natural selection would therefore favour the ability to recognise the intensity of perceived costs [10], and animals would exhibit appropriate behaviour for a given type and intensity of each perceived cost [53]. Based on the field experiments, the redfin minnow's diel activity patterns suggest three probable adaptive mechanisms to both direct and indirect costs, such as predation and competition, respectively. First, by being active during the day, minnows could potentially capitalise on feeding efficiency that would be conferred by the light hours for both prey detection and capture while avoiding predation from nocturnal predators, such as longfin eel. Second, the shoaling behaviour by minnows that was observed in this study may suggest an adaptive mechanism to visual terrestrial predators. Shoaling behaviour in fishes has been observed to be an important anti-predator strategy in streams that are subject to high predation risk [54]. By shoaling, individuals benefit from increased vigilance, coordinated anti-predator responses and a dilution effect [55]–[57]. Third, our results indicated stronger depth-dependence for redfin abundance during the day compared to night. This suggests that although the redfin minnow was diurnal, it was more active in deeper pools, which could potentially curtail the risk of visual terrestrial predators, particularly shallow diving and wading birds.

Theory predicts that when populations are exposed to variable environments with reliable cues, selection favours adaptive plasticity [58]. For example, in predator-mediated environments, prey activity is considered to be inversely related to predation or risk intensity [59]. It is therefore anticipated that removing potential predator cues through controlled laboratory experiments would induce different behavioural responses by prey. The results of our laboratory experiments, nonetheless, did not show any major shifts that indicated an innate recognition of predator absence. The results for these two species are therefore consistent with the suggestion that animals, particularly prey species, have evolved inherent behaviours that indicate anticipated rather than observed risk [26]. For chubbyhead barbs, their consistent nocturnal activity based on both field and laboratory observations suggests that their behaviour was a response to an anticipated risk associated with diurnal activity. Similar studies on aquatic invertebrates have shown that taxa that have evolved nocturnal habits in response to diurnal predators remain nocturnal in the absence of the predator [60]. In comparison, redfin minnows exhibited high refugia use during the diel cycle in the laboratory that appeared contrary to its high diurnal activity in the wild. This altered behaviour by redfin minnows may be explained by two probable mechanisms. First, the redfin minnows showed both depth-dependence and shoaling behaviour in the wild as possible predator avoidance mechanisms. Depth-dependence was, however, not simulated by our experiment where all animals were tested at a consistent depth of approximately 20 cm. Furthermore, our results showed that habitat association by redfin minnows was influenced by conspecifics, which suggests the importance of shoaling behaviour for the diurnal activity of this species. It is likely therefore that these fish would utilise refuge both at shallow depth and in the absence of big shoals that would potentially confer refugia advantage. Second, studies have shown that food availability plays an important role in the diel activity of fish. For example, experimental studies on European minnows *Phoxinus phoxinus* showed that they were likely to spend more time in refuge when they had abundant food supply [13]. Because the fish in the study were well-nourished, it is likely that they would utilise refuge during the day. This is consistent with the suggestion that fish would minimise exposure to diurnal predators by utilising refuge when their energetic requirements are satisfied [13]. The results of this study were also consistent with observations from other studies that suggest that when animals either regard their foraging area to be risky or have an imperfect knowledge about their environment, they tend to overestimate risk [26], [61], [62]. This often manifests in extensive refuge use or reduced foraging time [26], as was observed for the redfin minnows. Despite their high refuge use during the diel cycle, the redfin minnows showed high diurnal activity that was consistent with observations in the wild.

The diel activity patterns for both species were influenced by habitat structure. Physical habitat has been found to affect daily activity patterns in fish [47]. Often, for prey fishes, habitats that are profitable for foraging may not be optimal for refuge. Individuals would therefore select habitats that provide a good trade-off between energy intake and risk avoidance [14], [63]. In streams, physical refugia that provide cover from predators or habitats in which predators are inefficient often provide such trade-offs [47]. In this study, we found significant associations between fish abundance and coarse substratum (boulders and cobbles) in the wild, whereas in the laboratory the fish tended to show avoidance of open-water by utilising the concealed pipe refugia. Furthermore, our field observations indicated low fish abundance in fine substratum, such as gravel, which suggests that habitats dominated by this substratum were suboptimal and were avoided. This suggests that diel activity was mediated by habitat complexity. In particular, the interstitial spaces associated with coarse substrates not only provide refuge, but are also associated with invertebrate prey for fish [64].

### Implications on biological invasions

Non-native piscivorous fish are considered to be the major threat to both populations for these minnows [65]. Although most populations for both species occur in headwater streams, periodic incursions by the non-native fishes into these sections have been reported [66]. The probable invasion pathways include deliberate illegal introductions by anglers, especially for largemouth and smallmouth bass, and through movement from mainstem sections into headwater streams when habitats become connected during periods of high flow (particularly for sharptooth catfish).

Previous studies on predation impact have shown local extirpations of redfin minnows in habitats invaded by largemouth and smallmouth bass and trout that are known to be visual predators [67], [68]. By comparison, chubbyhead barbs occur in sympatry with trout and bass in certain habitats where they have been deliberately stocked as fodder fish (Booth pers. obs). The nocturnal habits of chubbyhead barbs suggest a pre-adaptive response to potential predation by diurnal visual predators. This nocturnal behaviour may explain, in part, the co-occurrence of the chubbyhead barbs with non-native predators, such as bass and trout. Nevertheless, it is unclear whether such co-occurrences are associated with both predation and non-consumptive costs. In addition, potential invasion by sharptooth catfish, which is now dominant in the mainstem sections of many rivers in the region [33], [66], may offset this prior advantage as it is known to have nocturnal habits [69].

The diurnal activity of redfin minnows may, also in part, explain its vulnerability to visual predators. Although we observed its shoaling behaviour as a potential anti-predator mechanism, such behaviour may have also evolved in response to a known visual predator, such as birds, and may therefore be an inappropriate behaviour if exposed to a novel aquatic predator. This shoaling behaviour may increase vulnerability to visual predators, such as bass and trout, as observed in predation impact studies (e.g. [67]). Some studies suggest that when prey species learn to recognise novel predators, they respond by either altering their diel activity [70] or they shift their habitat use by moving to shallow habitats to avoid predation by non-native piscivores [71]. Behavioural modifications by native prey species in response to the presence of non-native predators may nonetheless be associated with non-consumptive effects, such as use of suboptimal habitats and limited foraging time that would have an effect on population fitness [22], whereas, shifting habitat use could expose these fishes to terrestrial prey [72]. The diurnal activity and shoaling behaviour of redfin minnows may explain why this species has experienced severe localised extirpations in river sections that have been invaded by visual predators, particularly *Micropterus* spp. that are known to be active during day time periods.

## Supporting Information

Table S1 Summary of GLMMs based on the best-subset model describing the abundance patterns of chubbyhead barb *Barbus anoplus* and Eastern Cape redfin minnow *Pseudobarbus afer* that were captured in the wild in relation to photoperiod and physical habitat variables.
(DOC)

Table S2 Summary of GLMMs and LMMs on laboratory observations based on the best subset model describing either the frequency of observations or activity levels within different refugia for chubbyhead barb *Barbus anoplus* and Eastern Cape redfin minnow *Pseudobarbus afer* in relation to photoperiod, choice of refuge, size, presence of conspecifics.
(DOC)

## References

[1] Cohen R, Kronfeld-Schor N (2006) Individual variability and photic entrainment of circadian rhythms in golden spiny mice. Physiol Behav 87: 563–574. 16457859 10.1016/j.physbeh.2005.12.010

[2] Wasserberg G, Kotler BP, Abramsky Z (2006) The role of site, habitat, seasonality and competition in determining the nightly activity patterns of psammophilic gerbils in a centrifugally organized community. Oikos 112: 573–579.

[3] Levy O, Dayan T, Kronfeld-Schor N (2007) The relationship between the golden spiny mouse circadian system and its diurnal activity: An experimental field enclosures and laboratory study. Chronobiol Int 24: 599–613. 17701675 10.1080/07420520701534640

[4] Stuble KL, Rodriguez-Cabal MA, McCormick GL, Jurić I, Dunn RR, et al (2013) Tradeoffs, competition, and coexistence in eastern deciduous forest ant communities. Oecologia 171: 981–992. 23242423 10.1007/s00442-012-2459-9

[5] Kronfeld-Schor N, Dayan T (2003) Partitioning of time as an ecological resource. Annu Rev Ecol Evol Syst 34: 153–181.

[6] Jones M, Mandelik Y, Dayan T (2001) Coexistence of temporally partitioned spiny mice: roles of habitat structure and foraging behavior. Ecology 82: 2164–2176.

[7] Gutman R, Dayan T (2005) Temporal partitioning: an experiment with two species of spiny mice. Ecology 86: 164–173.

[8] Harrington LA, Harrington AL, Yamaguchi N, Thom MD, Ferreras P, et al (2009) The impact of native competitors on an alien invasive: temporal niche shifts to avoid interspecific aggression. Ecology 90: 1207–1216. 19537542 10.1890/08-0302.1

[9] Brook LA, Johnson CN, Ritchie EG (2012) Effects of predator control on behaviour of an apex predator and indirect consequences for mesopredator suppression. J Appl Ecol 49: 1278–1286.

[10] Helfman GS (1993) Fish behaviour by day, night and twilight. In: Pitcher TJ, editor. The Behaviour of Teleost Fishes, 2nd edn. London: Chapman & Hall. pp. 479–512.

[11] Fraser NHC, Metcalfe NB (1997) The costs of becoming nocturnal: feeding efficiency in relation to light intensity in juvenile Atlantic salmon. Funct Ecol 11: 385–91.

[12] Power ME (1984) Depth distributions of armoured catfish: predator-induced resource avoidance? Ecology 65: 523–528.

[13] Metcalfe NB, Steele GI (2001) Changing nutritional status causes a shift in the balance of nocturnal to diurnal activity in European minnows. Funct Ecol 15: 304–309.

[14] Railsback SF, Harvey BC, Hayse JW, LaGory KE (2005) Tests of theory for diel variation in salmonid feeding activity and habitat use. Ecology 86: 947–959.

[15] Alanara A, Burns MD, Metcalfe NB (2001) Intraspecific resource partitioning in brown trout: the temporal distribution of foraging is determined by social rank. J Anim Ecol 70: 980–986.

[16] Crook DA, Robertson AI, King AJ, Humphries P (2001) The influence of spatial scale and habitat arrangement on diel patterns of habitat use by two lowland river fishes. Oecologia 129: 525–533. 24577692 10.1007/s004420100750

[17] Clavero M, Blanco-Garrido F, Zamora L, Prenda J (2005) Size-related and diel variations in microhabitat use of three endangered small fishes in a Mediterranean coastal stream. J Fish Biol 67: 72–85.

[18] Kramer DL, Rangeley RW, Chapman LJ (1997) Habitat selection: patterns of spatial distribution from behavioural decisions. In: Godin JJ, editor. Behavioural ecology of teleost fishes. Oxford: Oxford University Press. pp. 37–80.

[19] Ayala JR, Rader RB, Belk MC, Schaalje BG (2007) Ground-truthing the impact of invasive species: spatio-temporal overlap between native least chub and introduced western mosquitofish. Biol Invas 9: 857–869.

[20] Correa C, Hendry AP (2012) Invasive salmonids and lake order interact in the decline of puye grande *Galaxias platei* in western Patagonia lakes. Ecol Appl 22: 828–842. 22645814 10.1890/11-1174.1

[21] Miller SA, Gunckel S, Jacobs S, Warren D (2013) Sympatric relationship between redband trout and non-native brook trout in the Southeastern Oregon Great Basin. Environ Biol Fish (Early online) DOI 10.1007/s10641-013-0157-z.

[22] Havird JC, Weeks JR, Hau S, Santos S (2013) Invasive fishes in the Hawaiian anchialine ecosystem: investigating potential predator avoidance by endemic organisms. Hydrobiologia 716: 189–201.

[23] Metcalfe NB, Fraser NHC, Burns MD (1998) State-dependent shifts between nocturnal and diurnal activity in salmon. P Roy Soc Lond B 265: 1503–1507.

[24] Kronfeld-Schor N, Dayan T, Elvert R, Haim A, Zisapel N, et al (2001) On the use of the time axis for ecological separation: diel rhythms as an evolutionary constraint. Am Nat 158: 451–457. 18707339 10.1086/321991

[25] Roll U, Dayan T (2002) Family ties and activity time in the order Rodentia. Isr J Zool 48: 177–78.

[26] Metcalfe NB, Fraser NHC, Burns MD (1999) Food availability and the nocturnal vs. diurnal foraging trade-off in juvenile salmon. J Anim Ecol 68: 371–381.

[27] Halle S (2006) Polyphasic activity patterns in small mammals. Folia Primatol 77: 15–26. 16415575 10.1159/000089693

[28] Schoener TW (1986) Resource partitioning. In: Kikkawa J, Anderson DJ, editors. Community ecology: pattern and process. California: Blackwell, Palo Alto. pp. 91–126.

[29] Payne NL, van der Meulen DE, Gannon R, Semmens JM, Suthers IM, et al (2012) Rain reverses diel activity rhythms in an estuarine teleost. Proc R Soc B 280: 2012–2363. 10.1098/rspb.2012.2363 PMC3574447 23173211

[30] Reebs SG (2002) Plasticity of diel and circadian activity rhythms in fishes. Rev Fish Biol Fish 12: 349–371.

[31] Fox RJ, Bellwood DR (2011) Unconstrained by the clock? Plasticity of diel activity rhythm in a tropical reef fish, *Siganus lineatus* . Funct Ecol 25: 1096–1105.

[32] Schoener TW (1974) Resource partitioning in ecological communities. Science 185: 27–38. 17779277 10.1126/science.185.4145.27

[33] Kadye WT, Booth AJ (2013) An invader within an altered landscape: one catfish, two rivers and an inter-basin water transfer scheme. River Res Appl 29: 1131–1146.

[34] Skelton P (2001) A complete guide to the freshwater fishes of southern Africa. Cape Town: Struik Publishers.

[35] Kadye WT, Booth AJ (2012) Inter-seasonal persistence and size-structuring of two minnow species within headwater streams in the Eastern Cape, South Africa. J Appl Ichthyol 28: 791–799.

[36] IUCN (2013) IUCN Red List of Threatened Species. Version 2013.1.Online at: http://www.iucnredlist.org (accessed 13 November 2013).

[37] Bovee KD (1986) Development and evaluation of habitat suitability criteria for use in the instream flow incremental methodology. Fort Collins, CO, U.S. Fish and Wildlife Service Biological Report 86(7).

[38] Cambray JA, Bruton MN (1985) Age and growth of a colonizing minnow, *Barbus anoplus*, in a man-made lake in South Africa. Environ Biol Fish 12: 131–141.

[39] Cambray JA, Hecht T (1995) Comparison of the growth of two closely related redfin minnows, *Pseudobarbus afer* (Peters, 1864) and *P. asper* (Boulenger, 1911) (Pisces, Cyprinidae), in the Gamtoos River System, South Africa. J Afr Zool 109: 350–376.

[40] Bolker BM, Brooks ME, Clark CJ, Geange SW, Poulsen JR, et al (2009) Generalized linear mixed models: a practical guide for ecology and evolution. Trends Ecol Evol 24: 127–135. 19185386 10.1016/j.tree.2008.10.008

[41] Lin DY, Wei LJ, Ying Z (2002) Model-checking techniques based on cumulative residuals. Biometrics 58: 1–12. 11890304 10.1111/j.0006-341x.2002.00001.x

[42] Burnham KP, Anderson DR (2002) Model selection and multimodel inference: a practical information-theoretic approach. 2nd ed. New York: Springer-Verlag.

[43] R Development Core Team (2013) R: A language and environment for statistical computing. Vienna: R Foundation for Statistical Computing, Vienna, Austria.ISBN 3-900051-07-0.

[44] Bates D, Maechler M, Bolker B (2011) lme4: Linear mixed-effects models using S4 classes. R package version 0.999375-41. http://CRAN.R-project.org/package=lme4.

[45] Barton K (2011) MuMIn: Multi-model inference. R package version 1.0.0. http://CRAN.R-project.org/package=MuMIn.

[46] Reebs SG, Boudreau L, Hardie R, Cunjak RA (1995) Diel activity patterns of lake chubs and other fishes in a temperate stream. Can J Zool 73: 1221–1227.

[47] Bradford MJ, Higgins PS (2001) Habitat-, season-, and size-specific variation in diel activity patterns of juvenile chinook salmon (*Oncorhynchus tshawytscha*) and steelhead trout (*Oncorhynchus mykiss*). Can J Fish Aquat Sci 58: 365–374.

[48] Fraser DF, Gilliam JF, Akkara JT, Albanese BW, Snider SB (2004) Night feeding by guppies under predator release: effects on growth and daytime courtship. Ecology 85: 312–319.

[49] Tikkanen P, Muotka T, Huhta A (1994) Predator detection and avoidance by lotic mayfly nymphs of different size. Oecologia 99: 252–259. 28313879 10.1007/BF00627737

[50] Baker RL, Ball SL (1995) Microhabitat selection by larval *Chironomus tentans* (Diptera, Chironomidae)-effects of predators, food, cover and light. Freshwater Biol 34: 101–106.

[51] Lampert W (1989) The adaptive significance of diel vertical migration of zooplankton. Funct Ecol 3: 21–27.

[52] Lima SL, Bednekoff PA (1999) Temporal variation in danger drives antipredator behavior: the predation risk allocation hypothesis. Am Nat 153: 649–659. 29585647 10.1086/303202

[53] Botham MS, Hayward RK, Morrell LJ, Croft DP, Ward JR, et al (2008) Risk-sensitive antipredator behavior in the Trinidadian guppy *Poecilia reticulata* . Ecology 89: 3174–3185. 10.1890/07-0490.1 31766795

[54] Queiroz H, Magurran AE (2005) Safety in numbers? Shoaling behaviour of the Amazonian red-bellied piranha. Biol Lett 1: 155–157. 17148153 10.1098/rsbl.2004.0267 PMC1626212

[55] Foster WA, Treherne JE (1981) Evidence of dilution effect in the selfish herd from fish predation on a marine insect. Nature 293: 466–467.

[56] Krause J, Ruxton GD (2002) Living in groups. Oxford: Oxford University Press. 228 p.

[57] Orpwood JE, Magurran AE, Armstrong JD, Griffiths SW (2008) Minnows and the selfish herd: effects of predation risk on shoaling behaviour are dependent on habitat complexity. Anim Behav 76: 143–152.

[58] Agrawal AA (2001) Phenotypic plasticity in the interactions and evolution of species. Science 294: 321–326. 11598291 10.1126/science.1060701

[59] Relyea RA (2004) Fine-tuned phenotypes: tadpole plasticity under 16 combinations of predators and competitors. Ecology 85: 172–179.

[60] Flecker AS (1992) Fish predation and the evolution of invertebrate drift periodicity evidence from neotropical streams. Ecology 73: 438–448.

[61] Bouskila A, Blumstein DT (1992) Rules of thumb for predation hazard assessment predictions from a dynamic model. Am Nat 139: 161–176.

[62] Bouskila A (1995) Interactions between predation risk and competition a field-study of kangaroo rats and snakes. Ecology 76: 165–178.

[63] Dupuch A, Magnan P, Bertolo A, Dill LM, Proulx M (2009) Does predation risk influence habitat use by northern redbelly dace *Phoxinus eos* at different spatial scales? J Fish Biol 74: 1371–1382. 20735640 10.1111/j.1095-8649.2009.02183.x

[64] Mueller R, Pyron M (2010) Fish assemblages and substrates in the Middle Wabash River, USA. Copeia 1: 47–53.

[65] Tweddle D, Bills R, Swartz E, Coetzer W, Da Costa L, et al.. (2009) The status and distribution of freshwater fishes. In: Darwall WR, Smith KG, Tweddle D, Skelton PH, editors. The status and distribution of freshwater biodiversity in southern Africa. Gland (Switzerland) and Grahamstown (South Africa): IUCN and South African Institute for Aquatic Biodiversity. pp. 21–37.

[66] Ellender BR, Weyl OLF, Swartz ER (2011) Invasion of a headwater stream by non-native fishes in the Swartkops River system, South Africa. Afr Zool 46: 39–46.

[67] Lowe SR, Woodford DJ, Impson DN, Day JA (2008) The impact of invasive fish and invasive riparian plants on the invertebrate fauna of the Rondegat River, Cape Floristic Region, South Africa. Afr J Aquat Sci 33: 51–62.

[68] Russell AI (2011) Conservation status and distribution of freshwater fishes in South African national parks. Afr Zool 46: 117–132.

[69] Bruton MN (1979) The food and feeding behaviour of *Clarias gariepinus* (Pisces: Clariidae) in Lake Sibaya, South Africa, with emphasis on its role as a predator of cichlids. Trans Zool Soc Lond 35: 47–114.

[70] Bool JD, Witcomb K, Kydd E, Brown C (2011) Learned recognition and avoidance of invasive mosquitofish by the shrimp, *Paratya australiensis* . Mar Fresh Res 62: 1230–1236.

[71] Schlosser IJ (1987) The role of predation in age- and size-related habitat use by stream fishes. Ecology 68: 651–659.

[72] Loppnow GL, Vascotto K, Venturelli PA (2013) Invasive smallmouth bass (*Micropterus dolomieu*): history, impacts and control. Manage Biol Invas 3: 191–206.

