# Supplementary material for: Alternative Responses to Predation in Two Headwater Stream Minnows Is Reflected in Their Contrasting Diel Activity Patterns
Source: PLoS One. 2014 Apr 1;9(4):e93666. doi: 10.1371/journal.pone.0093666 (PMC3972241; doi:10.1371/journal.pone.0093666)
Supplement: Table S1 — Summary of GLMMs based on the best-subset model describing the abundance patterns of chubbyhead barb Barbus anoplus and Eastern Cape redfin minnow Pseudobarbus afer that were captured in the wild in relation to photoperiod and physical habitat variables. (DOC) [file pone.0093666.s001.doc]

Table S1: Summary of GLMMs based on the best-subset model describing the abundance patterns of chubbyhead barb *Barbus anoplus* and Eastern Cape redfin minnow *Pseudobarbus afer* that were captured in the wild in relation to photoperiod and physical habitat variables.

|  | *Barbus anoplus* | | | |  | *Pseudobarbus afer* | | | |
| --- | --- | --- | --- | --- | --- | --- | --- | --- | --- |
|  | Estimate | SE | *z* | *P*(*z)* |  | Estimate | SE | *z* | *P*(*z)* |
| Intercept | -3.40 | 0.39 | -8.72 | < 0.001 |  | -0.93 | 0.23 | -4.01 | < 0.001 |
| Depth | 0.01 | 0.00 | 2.17 | 0.03 |  | 0.05 | 0.00 | 11.08 | < 0.001 |
| Photoperiod | 1.61 | 0.14 | 11.14 | < 0.001 |  | -0.36 | 0.17 | -2.08 | 0.04 |
| Boulder | 1.18 | 0.36 | 3.31 | < 0.001 |  |  |  |  |  |
| Cobble | 0.65 | 0.35 | 1.84 | 0.07 |  | -0.28 | 0.13 | -2.27 | 0.02 |
| Gravel | -18.40 | 2945.00 | -0.01 | 1.00 |  | -1.31 | 0.24 | -5.48 | < 0.001 |
| Vegetation | -0.23 | 0.43 | -0.52 | 0.60 |  |  |  |  |  |
| Photoperiod×Depth |  |  |  |  |  | -0.02 | 0.00 | -5.50 | < 0.001 |
| Boulder ×Vegetation | 0.25 | 0.50 | 0.50 | 0.61 |  |  |  |  |  |
| Cobble ×Vegetation | 0.57 | 0.46 | 1.25 | 0.21 |  |  |  |  |  |
| Gravel ×Vegetation | 17.48 | 2945.00 | 0.01 | 1.00 |  |  |  |  |  |
