# Supplementary material for: Alternative Responses to Predation in Two Headwater Stream Minnows Is Reflected in Their Contrasting Diel Activity Patterns
Source: PLoS One. 2014 Apr 1;9(4):e93666. doi: 10.1371/journal.pone.0093666 (PMC3972241; doi:10.1371/journal.pone.0093666)
Supplement: Table S2 — Summary of GLMMs and LMMs on laboratory observations based on the best subset model describing either the frequency of observations or activity levels within different refugia for chubbyhead barb Barbus anoplus and Eastern Cape redfin minnow Pseudobarbus afer in relation to photoperiod, choice of refuge, size, presence of conspecifics. (DOC) [file pone.0093666.s002.doc]

Table S2: Summary of GLMMs and LMMs on laboratory observations based on the best subset model describing either the frequency of observations or activity levels within different refugia for chubbyhead barb *Barbus anoplus* and Eastern Cape redfin minnow *Pseudobarbus afer* in relation to photoperiod, choice of refuge, size, presence of conspecifics.

|  |  | *Barbus anoplus* | | | |  | *Pseudobarbus afer* | | | |
| --- | --- | --- | --- | --- | --- | --- | --- | --- | --- | --- |
|  |  | Estimate | SE | *z* | *P*(*z*) |  | Estimate | SE | *z* | *P*(*z)* |
| Frequency | Intercept | 0.00 | 0.13 | 0.00 | 1.00 |  | 0.01 | 0.07 | 0.07 | 0.94 |
|  | Open water | 0.00 | 0.15 | 0.00 | 1.00 |  | -0.17 | 0.09 | -1.86 | 0.06 |
|  | Pipe | 0.00 | 0.25 | 0.00 | 1.00 |  | 0.02 | 0.06 | 0.27 | 0.79 |
|  | Conspecifics | 0.27 | 0.16 | 1.72 | 0.09 |  | 0.54 | 0.07 | 8.31 | < 0.001 |
|  | Photoperiod | 0.00 | 0.18 | 0.00 | 1.00 |  |  |  |  |  |
|  | Open water × Conspecifics | 0.55 | 0.18 | 3.05 | < 0.01 |  |  |  |  |  |
|  | Pipe × Conspecifics | -0.08 | 0.29 | -0.27 | 0.79 |  |  |  |  |  |
|  | Open water × Photoperiod | 0.00 | 0.33 | 0.00 | 1.00 |  |  |  |  |  |
|  | Pipe × Photoperiod | 0.00 | 0.29 | 0.00 | 1.00 |  |  |  |  |  |
|  | Conspecifics × Photoperiod | 0.23 | 0.22 | 1.04 | 0.30 |  |  |  |  |  |
|  | Open water × Conspecifics × Photoperiod | -0.64 | 0.68 | -0.95 | 0.34 |  |  |  |  |  |
|  | Pipe × Conspecifics × Photoperiod | 0.53 | 0.34 | 1.58 | 0.11 |  |  |  |  |  |
|  |  |  |  |  |  |  |  |  |  |  |
|  |  | Estimate | SE | *z* | *P*(*z*) |  | Estimate | SE | *z* | *P*(*z)* |
| Activity | Intercept | 2.94 | 0.01 | 199.59 |  |  | 2.17 | 0.06 | 35.21 |  |
|  | Photoperiod | -1.00 | 0.02 | -51.61 |  |  | 0.18 | 0.05 | 3.34 |  |
|  | Conspecifics |  |  |  |  |  | 0.45 | 0.08 | 5.70 |  |
